# Cerebral amyloid angiopathy-related inflammation presenting with steroid-responsive higher brain dysfunction: case report and review of the literature

**DOI:** 10.1186/1742-2094-8-116

**Published:** 2011-09-14

**Authors:** Hideya Sakaguchi, Akihiko Ueda, Takayuki Kosaka, Satoshi Yamashita, En Kimura, Taro Yamashita, Yasushi Maeda, Teruyuki Hirano, Makoto Uchino

**Affiliations:** 1 Department of Neurology, Faculty of Life Sciences, Kumamoto University 1-1-1 Honjo, Kumamoto 860-0811, Japan

**Keywords:** cerebral amyloid angiopathy, CAA-related inflammation, higher brain dysfunction

## Abstract

A 56-year-old man noticed discomfort in his left lower limb, followed by convulsion and numbness in the same area. Magnetic resonance imaging (MRI) showed white matter lesions in the right parietal lobe accompanied by leptomeningeal or leptomeningeal and cortical post-contrast enhancement along the parietal sulci. The patient also exhibited higher brain dysfunction corresponding with the lesions on MRI. Histological pathology disclosed β-amyloid in the blood vessels and perivascular inflammation, which highlights the diagnosis of cerebral amyloid angiopathy (CAA)-related inflammation. Pulse steroid therapy was so effective that clinical and radiological findings immediately improved.

CAA-related inflammation is a rare disease, defined by the deposition of amyloid proteins within the leptomeningeal and cortical arteries associated with vasculitis or perivasculitis. Here we report a patient with CAA-related inflammation who showed higher brain dysfunction that improved with steroid therapy. In cases with atypical radiological lesions like our case, cerebral biopsy with histological confirmation remains necessary for an accurate diagnosis.

## Background

Cerebral amyloid angiopathy (CAA) is a common pathology in the elderly characterized by the deposition of amyloid proteins within the leptomeningeal and cortical arteries [1]. Recently, coexisting inflammations in CAA patients, such as vasculitis or perivasculitis, which clinically resemble central nervous system vasculitis, have been recognized as CAA-related inflammation [2,3]. The inflammation typically responds well to steroid therapy [4], and recent studies have pointed out its similarities with meningoencephalitis induced by immunization to Aβ in Alzheimer disease patients [4-6]. Herein we report a patient with CAA-related inflammation who showed convulsion in the left lower extremity and higher brain dysfunction; both were dramatically improved by steroid therapy.

## Case presentation

**Figure 1:**
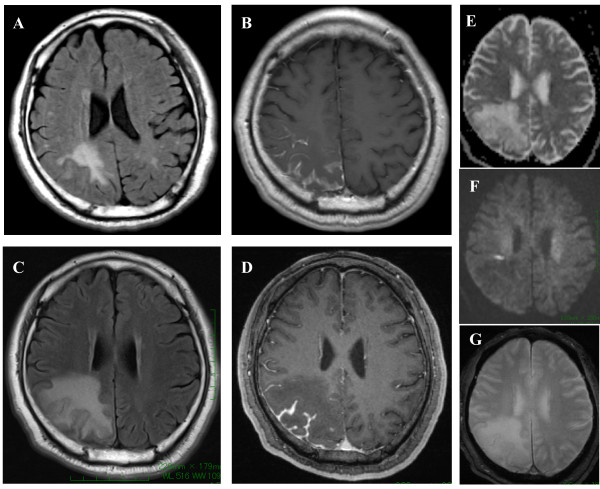
**Axial MRI from the referring hospital and on admission to our hospital**. MRI findings of FLAIR (A) and T1-weighted image with Gd enhancement (B) from the referring hospital (1.5T). Increased white matter lesions are visible in the right parietal lobe on FLAIR images (A), and a T1-weighted Gd enhanced image revealed abnormal enhanced parenchymal lesions along the parietal sulci (B). On admission, these lesions worsened in both FLAIR (C) and T1-weighted enhanced images (D). High signal intensity in the apparent diffusion coefficient (ADC) map (E) and low signal intensity in the diffusion-weighted image (F) suggested its edematous nature. No microhemorrhages were observed with Gradient recalled echo-T2* imaging (3T) (G).

A 56-year-old man first noticed discomfort in his left lower limb in January 2010. After 7 days, convulsion in the left lower limb suddenly occurred, and he was transported to the emergency hospital. Magnetic resonance imaging (MRI) showed increased white matter intensities in the right parietal lobe on T2-weighted and fluid attenuated inversion-recovery (FLAIR) images. T1-weighted gadolinium (Gd)-enhanced images revealed enhanced leptomeningeal lesions along the parietal sulci (Figure 1A-B). No microhemorrhages were observed with Gradient-recalled echo (GRE)-T2* imaging (1.5T). He was referred to our institution.

On admission, neurological exam showed mild hyperesthesia in the left lower limb and mild hypalgesia in the left crus. No other abnormal findings were present. Biochemical screening tests were generally normal except for serum C-reactive protein (0.77 mg/dL), soluble interleukin-2 receptor antibody (462 U/mL), erythrocyte sedimentation rate (26/1 h, 72/2 h), and carcinoembryonic antigen (4.5 ng/mL). In the cerebrospinal fluid, protein levels were elevated (72 mg/dl) and the cell count was mildly elevated (12/μL).

**Figure 2:**
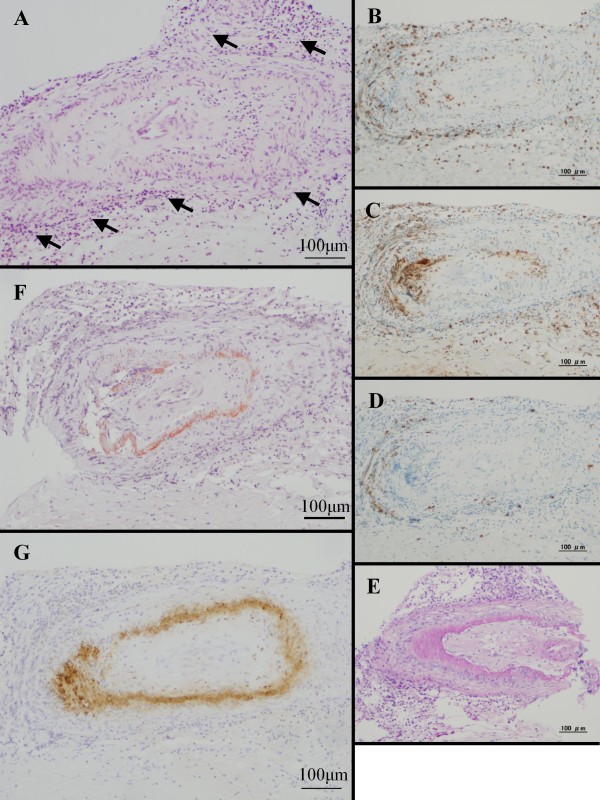
**Histological and immune-histological examination of brain biopsy**. Microscopic examination showed nonspecific meningoencephalitis involving perivasculitis of leptomeninges (arrows) and cortical gray matter (A). The cellular infiltrate was mainly composed of CD-3-positive T-lymphocytes (B) and CD-68-positive macrophages (C) with minimal CD-20-positive B-lymphocytes (D). PAS staining showed no deposits (E). Congo-red staining revealed amyloid positive blood vessels (F); the amyloid was disclosed to be amyloid-β by immunohistochemical staining (G).

Because a follow-up MRI revealed progression of the white matter lesions and parenchymal enhanced lesions without microhemorrhages (GRE-T2* imaging; 3T) (Figure 1C-G), a brain biopsy was performed in March 2010. Histological pathology showed nonspecific meningoencephalitis involving perivasculitis of the leptomeninges and cortical gray matter (Figure 2A-D).

Starting in April 2010, the patient complained of difficulty with his handwriting. Neuropsychological tests of higher brain functions revealed mild constructional apraxia, line imbalance for words and numbers, difficulty drawing a figure following oral instructions, and problems with visual reproduction. No apathy or dementia was observed.

After the episode, further histological analysis with Congo-red staining disclosed amyloid laden blood vessels. Immunohistochemical staining for β-amyloid led to the diagnosis of CAA-related inflammation (Figure 2F-G).

**Figure 3:**
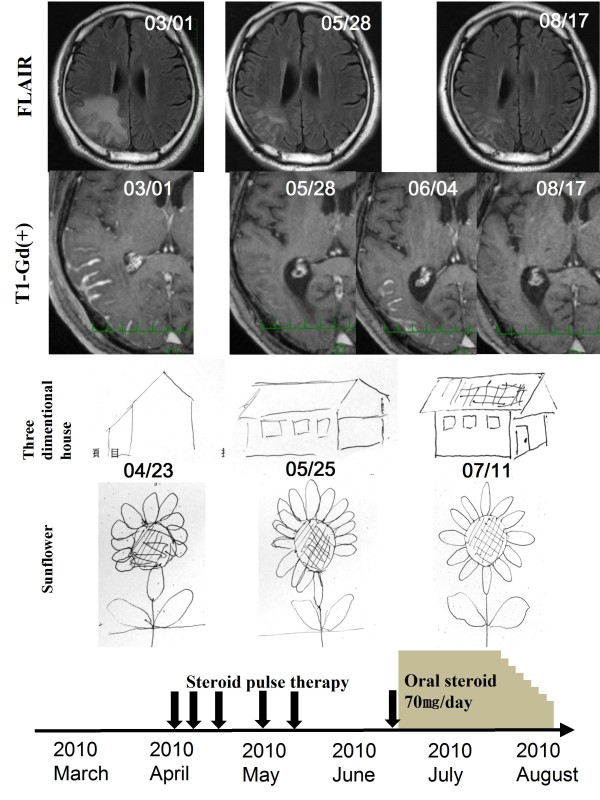
**Clinical course of treatment with steroid**. Abnormal T1 Gd-enhanced findings immediately improved in the fifth course of steroid pulse therapy, accompanied by a gradual decrease of FLAIR findings and a gradual improvement in higher brain function. As the MRI lesions improved (05/28), the descriptions of the 3D-house and sunflower were made more vivid (05/25). Because T1 Gd-enhanced lesions almost disappeared after the fifth course of the steroid (05/28), we stopped the steroid therapy, and the lesion relapsed (06/04). However, after the initiation of oral steroid therapy, no relapse was observed either clinically or radiologically (08/17).

Steroid pulse therapy (methylprednisolone 1 g/day for 3 days) was performed. The abnormal Gd-enhanced findings immediately improved with gradually decreasing FLAIR findings, and the higher brain dysfunctions also gradually resolved (Figure 3).

After the fifth course of steroid pulse treatment, the T1-enhanced lesions had almost disappeared, and we stopped the treatment. However, 2 weeks later, the lesions had relapsed on a follow-up MRI, although no clinical signs were observed. We performed pulse steroid therapy again, followed by oral methylprednisolone therapy (70 mg/day). After the oral steroid therapy was initiated, no relapses were observed either clinically or radiologically. Two months later, the oral steroid was tapered at a rate of 5 mg/week, and he was discharged on a regimen of methylprednisolone 30 mg/day.

## Discussion

CAA is defined by the deposition of amyloid proteins within leptomeningeal and cortical arteries, arterioles, and capillaries [1]. Recently, a subset of patients who presented with seizures, subacute cognitive decline, or headaches with hyperintensities on T2-weighted or FLAIR MRI images with microhemorrhages were described as having CAA-related inflammation [2,3]. Neuropathologic examination has generally revealed angiitis of CAA-affected vessels and peripheral inflammation, presenting as vasculitis or perivasculitis [7]. Both pathologic forms can co-exist, and it has been suggested that the prognosis is better for the perivascular type [8]. This inflammation appears to represent an autoimmune response to vascular β-amyloid deposits. The mechanism by which this immune response occurs is not well understood, although one possible factor is the increased frequency of apolipoprotein E ε4/ε4 genotype [9].

The clinical spectrum of CAA-related inflammation is mainly composed of rapidly progressive dementia and seizure. Although the initial presentation of our case was seizure and numbness, the subsequent higher brain dysfunction is uncommon. To clarify how often higher brain dysfunction has been observed, we reviewed previous cases including our case (Table 1) [1,3,4,7-37]. In 64 cases, 10 presented with higher brain dysfunction without encephalopathy or dementia (15.3%). The most frequent symptom was aphasia (6 cases: 9.3%), followed by hemineglect (2 cases: 3.1%). One other case was reported of various higher brain dysfunction without mental change or dementia, like our case [23]. In these ten cases with higher brain dysfunction, MRI lesions and the presence of leptomeningeal enhancement were inconsistent, and thus the presentation of higher brain dysfunction was considered to be derived from the observed lesion rather than specific to CAA-related inflammation.

**Table 1:** Review of reported cases of CAA-related inflammation

Reference	n	Age	Sex	Clinical presentation	MRI lesion		Micro bleeds in T2*-weighted images	MRI enhanced lesion	Pathology	treatment	Outcome
Greenberg et al. 1993 [10]	1	72	F	dementia headache	left	frontal	NA	(-)	vasculitis	NA	NA

Ortiz et al. 1996 [11]	1	68	F	headache	right	temporal/parietal	NA	(-)	vasculitis	steroid	NA

Fountain et al. 1996 [12]	2	66	M	fluent aphasia right hemianopia	bilateral	temporal/parietal	NA	(-)	vasculitis perivasculitis	steroid cyclophosphamide	alive relapse (+)
		
		69	F	headache confusion focal neurology seizure	bilateral	confluent multifocal	NA	NA	vasculitis	steroid cyclophosphamide	died relapse (+)

Anders et al. 1997 [13]	2	70	M	mental status change	right	frontal	NA	NA	vasculitis	NA	NA
		
		69	M	headache lethargy behavior change	bilateral	white matter	NA	(+)	vasculitis	NA	NA

Fountain et al. 1999 [14]	1	71	M	headache confusion gait difficulty left hand apraxia	right	temporal/parietal	NA	NA	vasculitis	cyclophosphamide	alive relapse (+)

Scully et al. 2000 [15]	1	63	M	behavior change ataxia	bilateral	white matter	NA	(+)	perivasculitis	cyclophosphamide	alive

Oide et al. 2002 [16]	1	69	M	dizziness dementia seizure	bilateral	symmetrical periventricular	NA	NA	vasculitis	(-)	NA

Schwab et al. 2003 [8]	2	74	M	seizure dementia headache	bilateral	multifocal	NA	(+)	perivasculitis	steroid	alive relapse (+)
		
		70	F	dementia headache	right	temporal	NA	(+)	perivasculitis	steroid	alive relapse (+)

Tamargo et al. 2003 [17]	1	80	F	dementia left-side hemineglect word finding difficulty	bilateral	left frontal right parietal	NA	(+)	vasculitis	steroid	alive

Oh et al. 2004 [1]	2	80	F	Headache aphasia	bilateral	right parietal/occipital left frontal	NA	(-)	perivasculitis	steroid	alive
		
		77	M	aphasia	left	temporal	NA	(-)	vasculitis	steroid	alive

Safriel et al. 2004 [18]	1	49	M	seizure	right	occipital/temporal	NA	(-)	vasculitis	steroid	alive

Hashizume et al. 2004 [19]	1	65	M	headache left hemianopsia left-side hemineglect	right	temporal/occipital	NA	(+)	vasculitis	steroid cyclophosphamide	died

Harkness et al. 2004 [20]	1	72	F	dementia	bilateral	frontal	NA	(-)	vasculitis	no specific therapy	alive

Jacobs et al. 2004 [21]	1	81	F	confusion Balint's syndrome agraphia right-left confusion finger anomia left-side neglect	bilateral	parietal/occipital	NA	(+)	vasculitis	steroid	alive

Scolding et al. 2005 [3	6	69.3*	M 3 F 3	encephalopathy 6 focal neurology 2 seizure 1 headache 2	NA	mutifocal 1 frontal 1 diffuse white matter 1 right occipital 1 left frontal 1 bilateral confluent 1	NA	(+) 1 (-) 5	vasculitis	steroid 3 steroid cyclophosphamide 2 tumor resection steroid 1	alive 4 (relapse NA) died 2

Mikolaenko et al. 2006 [22]	1	50	M	seizure	right	frontal	NA	(+)	vasculitis	surgery	alive

Wong et al. 2006 [23]	1	79	F	higher brain dysfunction fatigue	right	frontal/temporal/parietal	NA	NA	vasculitis	steroid	alive relapse (+)

Kinnecom et al. 2007 [4]	1	62.3*	M 9 F 3	encephalopathy 9 headache 5 seizure 7 aphasia 1 presyncope 1	NA	NA	NA (the presence of microbleeds are mentioned but the proportion is not mentioned)	NA	perivasculitis	steroid 9 steroid cyclophosphamide 3	alive 11 (relapse (+) 3) died 1

Greenberg et al. 2007 [24]	1	63	M	headache behavioral change cognitive change	bilateral	multiple	NA	(+)	vasculitis	cyclophosphamide	alive relapse (+)

Marotti et al. 2007 [25]	1	57	F	headache seizure	bilateral	frontal/temporal/insular right thalamus	(+)	(+)	vasculitis	seizure control	died

McHugh et al. 2007 [26]	1	80	F	confusion incontinent urine global aphasia seizure right hemianopia right hemiparesis	bilateral	frontal	(+)	(-)	vasculitis perivasculitis	steroid	alive relapse (+)

Takada et al. 2007 [27]	1	69	F	headache cognitive decline	bilateral	right frontal/parietal bilateral parietal/occipital	(+)	(-)	vasculitis	steroid	died

Machida et al. 2008 [28]	1	69	F	cognitive decline	bilateral	multifocal	(-)	(+)	perivasculitis	steroid	alive relapse (+)

Salvarani et al. 2008 [29]	8	63*	M6 F2	encephalopathy 6 focal neurology 2 headache 3 only aphasia with alexia 1	bilateral 8	multifocal	NA	(+) 5 (-) 3	vasculitis	steroid 3 steroid cyclophosphamide 5	improved 6 died 1 worsened 1

Amick et al. 2008 [30]	1	79	F	transient right sided weakness	left	occipital/parietal	NA	(-)	vasculitis	(-)	died

Alcalay et al. 2009 [31]	1	92	F	mental status change	bilateral	multifocal	(+)	(+)	(-)	steroid	alive

Daniëls et al. 2009 [32]	1	80	F	mental status change right sided hemiparesis dysphasia seizure	bilateral	left hemisphere right parietal/occipital	(+)	(-)	(-)	steroid	alive relapse (+)

Greenberg et al. 2010 [9]	1	87	F	seizure cognitive impairment	bilateral	multifocal	(+)	NA	perivasculitis	steroid	died

Kloppenborg et al. 2010 [7]	1	74	M	increased sleepiness loss of initiative seizure	bilateral	frontal	(+)	(+)	perivasculitis	steroid	alive

Morishige et al. 2010 [33]	1	78	F	motor aphasia dementia	left	frontal	NA	(+)	vasculitis	steroid	alive

Savoiardo et al. 2010 [34]	1	76	M	fatigue confusion	bilateral	temporal/occcipital/frontal	(+)	(-)	(-)	steroid	alive

Cano et al. 2010 [35]	1	76	M	transient motor aphasia transient headache	bilateral	temporal	(+)	NA	(-)	(-)	alive

DiFrancesco et al. 2011 [36]	1	68	M	memory loss mood disorder	bilateral	multifocal	(+)	(-)	NA	steroid	alive

Chung et al. 2011 [37]	3	83	F	seizure	bilateral	multifocal	NA	NA	vasculitis	steroid	died
		
		forties	F	headache mild hemiparesis sensory loss	right	parietal/occipital	(+)	NA	vasculitis	steroid	alive haemorrhage (+)
		
		72	M	seizure left-side neglect left hemianopia	bilateral	multifocal	NA	NA	vasculitis perivasculitis	steroid cyclophosphamide	died

our case	1	56	M	Seizure sensory disturbance higher brain dysfunction	right	parietal	(-)	(+)	perivasculitis	steroid	alive relapse (+)

From the literature, we extracted the cases of CAA-related inflammation in which an MRI was evaluated. If autopsy or biopsy was examined, the cases without inflammation were excluded. All cases satisfy the diagnostic criteria of definite or probable CAA-related inflammation proposed by Chung et al. [37]. In 64 cases, 10 presented with higher brain dysfunction without encephalopathy or dementia (15.3%). The most frequent symptom was aphasia (6 cases: 9.3%), followed by hemineglect (2 cases: 3.1%). One case besides the current presented with various higher brain dysfunction without mental change or dementia [23]. In these 10 cases with higher brain dysfunction, MRI lesions and the presence of leptomeningeal enhancement were inconsistent. Thirteen cases were examined with MRI with an echo gradient sequence, and microhemorrhages were not seen in 2 cases, including our case (13.3%).

The leptomeningeal enhancement status of 42 patients was mentioned, and the clinical courses of 39 patients were described. Only one patient among 19 patients with leptomeningeal enhancement died (5.3%); however, 7 of 20 patients without enhancement died (35%), suggesting that leptomeningeal enhancement might be a factor in good prognosis. *: calculated mean

The MRI presentation for CAA-related inflammation was previously described as characterized by large confluent areas of predominantly white matter hyperintense signal on T2-weighted or FLAIR images [34]. These lesions are typically asymmetric and involve one or more cortical lesions without evident preferential laterality. T2-weighted gradient-echo sequence images usually showed multiple scattered cortical or subcortical microhemorrhages [34]. However, these microhemorrhages were not observed in our case, resulting in a delayed diagnosis. In our review, 13 cases were examined by MRI with an echo gradient sequence, and microhemorrhages were not seen in 2 cases including our case (13.3%). A possible explanation is that the inflammation caused by the immunoreactivity to amyloid might precede the vascular change of cerebellar amyloid angiopathy in some cases, such that microhemorrhages were not observed in radiological exams. This suggests that the gradient-echo sequence image might not be adequate for diagnosis of CAA-related inflammation in all cases. Brain biopsy should be considered if CAA-related inflammation is highly suspected from clinical presentation, even if microhemorrhages were not radiologically observed.

Approximately three quarters of all patients described had a good clinical response to corticosteroid therapy. Additionally, patients presenting with CAA and meningeal enhancement seem to have less progressive disease [29]. In our review, the leptomeningeal enhancement status of 42 patients was mentioned, and the clinical courses of 39 patients were described. Among 19 patients with leptomeningeal enhancement, only one patient died (5.3%) and the remaining 18 patients survived. However, among the other 20 patients without enhancement, 7 patients died (35%), suggesting that leptomeningeal enhancement might be a good prognostic factor.

The distinctive pattern of asymmetric MRI lesions in CAA-related inflammation appears to be distinguishable from both non-inflammatory CAA and other causes. This observation raises the possibility that typical MRI findings should prove sufficient to diagnose CAA-related inflammation without necessitating brain biopsy [4]. However, in our case, preoperative imaging did not show the typical microhemorrhages associated with CAA, and the diagnosis could not have been established before biopsy. Therefore, we suggest that cerebral biopsy with histological confirmation remains necessary for an accurate diagnosis.

## Conclusion

We described a patient with CAA-related inflammation whose higher brain functions were dramatically improved by steroid therapy. Because the improvement of cognitive function paralleled resolution of the lesions seen on MRI, this report demonstrates clinically and radiologically progressive improvement of CAA-related inflammation. Our case also suggests the importance of brain biopsy for diagnosis in a case with atypical radiological findings, because correct diagnosis and treatment are crucial for successful recovery and good prognosis.

## Consent

Written informed consent was obtained from the patient for publication of this case report and any accompanying images. A copy of the written consent is available for review by the Editor-in-Chief of this journal.

## List of abbreviations

Aβ: amyloid β; ADC: apparent diffusion coefficient; CAA: cerebral amyloid angiopathy; FLAIR: fluid attenuated inversion-recovery; Gd: gadolinium; MRI: magnetic resonance imaging; GRE: gradient-recalled echo.

## Competing interests

The authors declare that they have no competing interests.

## Authors' contributions

HS designed this article and direction for investigations and drafted the manuscript. AU, TK, SY, EK, TY, YM, TH, and MU contributed to interpretations of clinical, radiological and pathological details. All authors read and approved the final manuscript.

## Authors' information

All authors are members of the Department of Neurology, Faculty of Life Sciences, Kumamoto University, and TK was also a graduate student of the Brain Research Institute, University of Niigata until March 2011.
